# Supplementary figures and images for: Enhanced hippocampal LTP but normal NMDA receptor and AMPA receptor function in a rat model of CDKL5 deficiency disorder
Source: Mol Autism. 2024 Jun 14;15:28. doi: 10.1186/s13229-024-00601-9 (PMC11177379; doi:10.1186/s13229-024-00601-9)

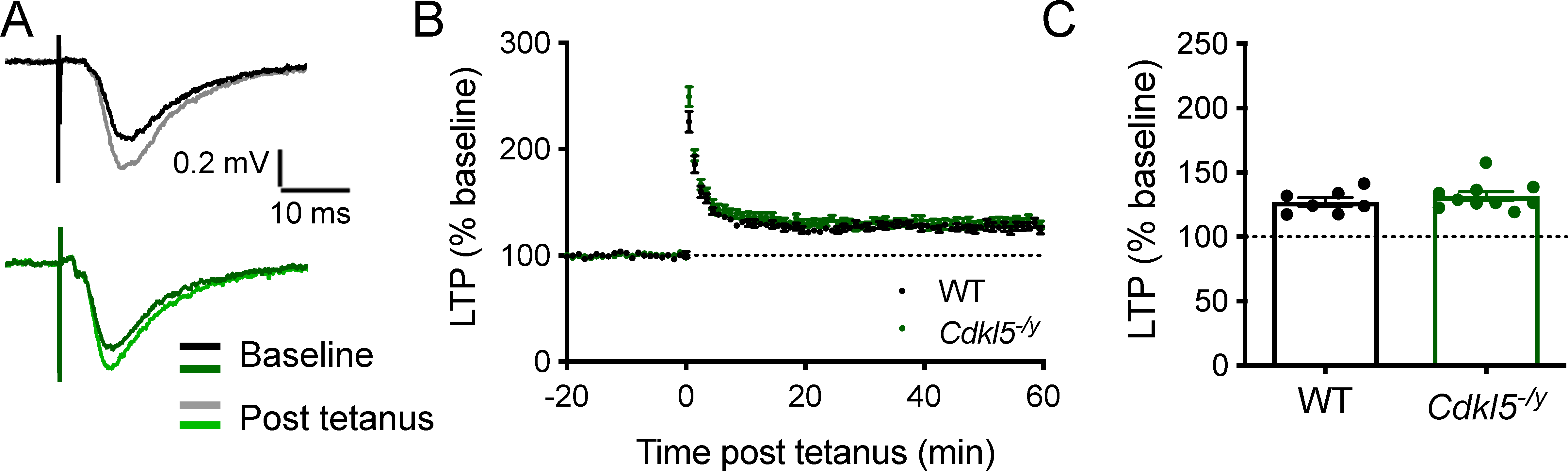

Supplement: Supplementary file 1 — Supplementary Material 1 [file 13229_2024_601_MOESM1_ESM.tif]

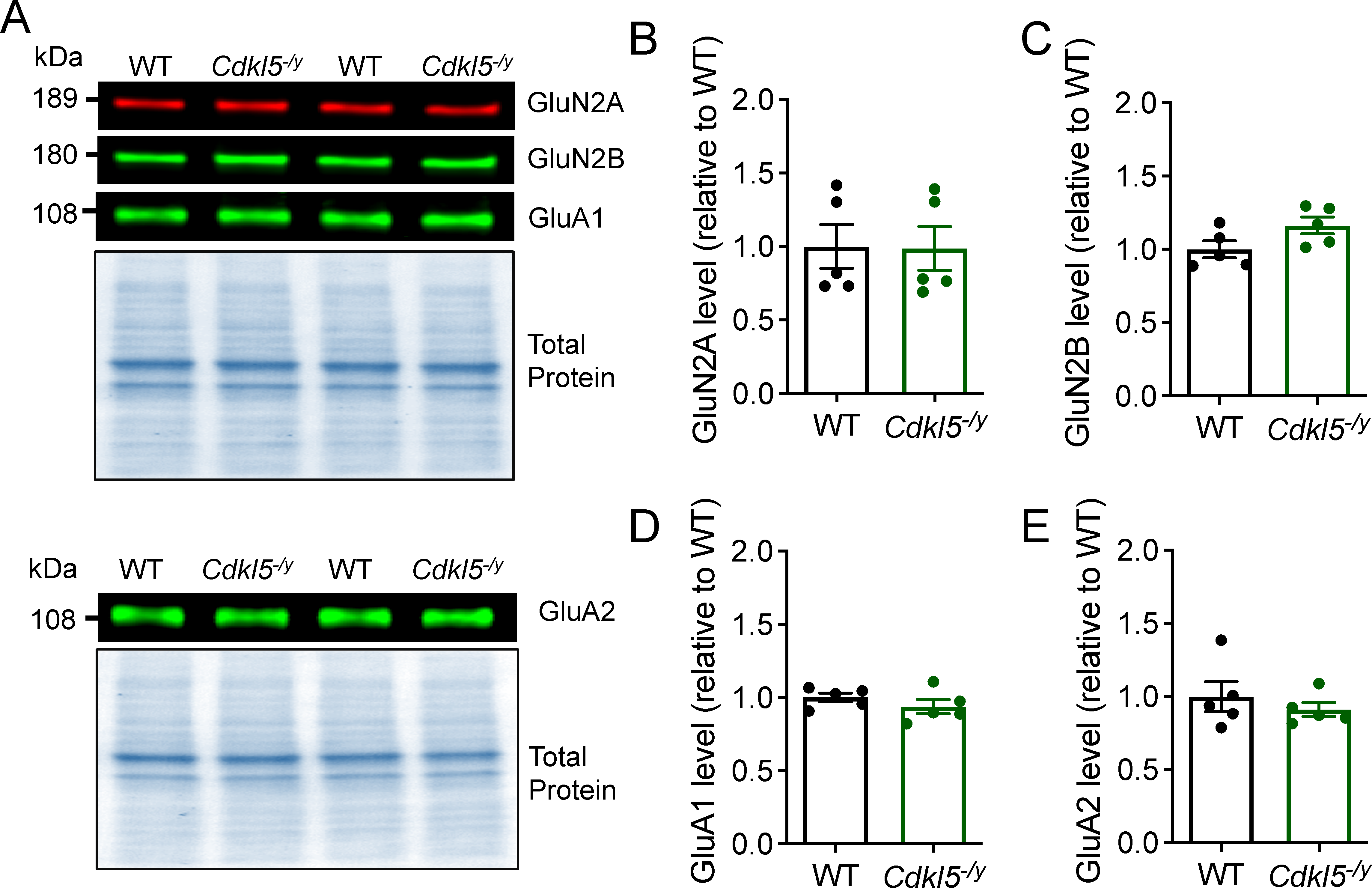

Supplement: Supplementary file 2 — Supplementary Material 2 [file 13229_2024_601_MOESM2_ESM.tif]

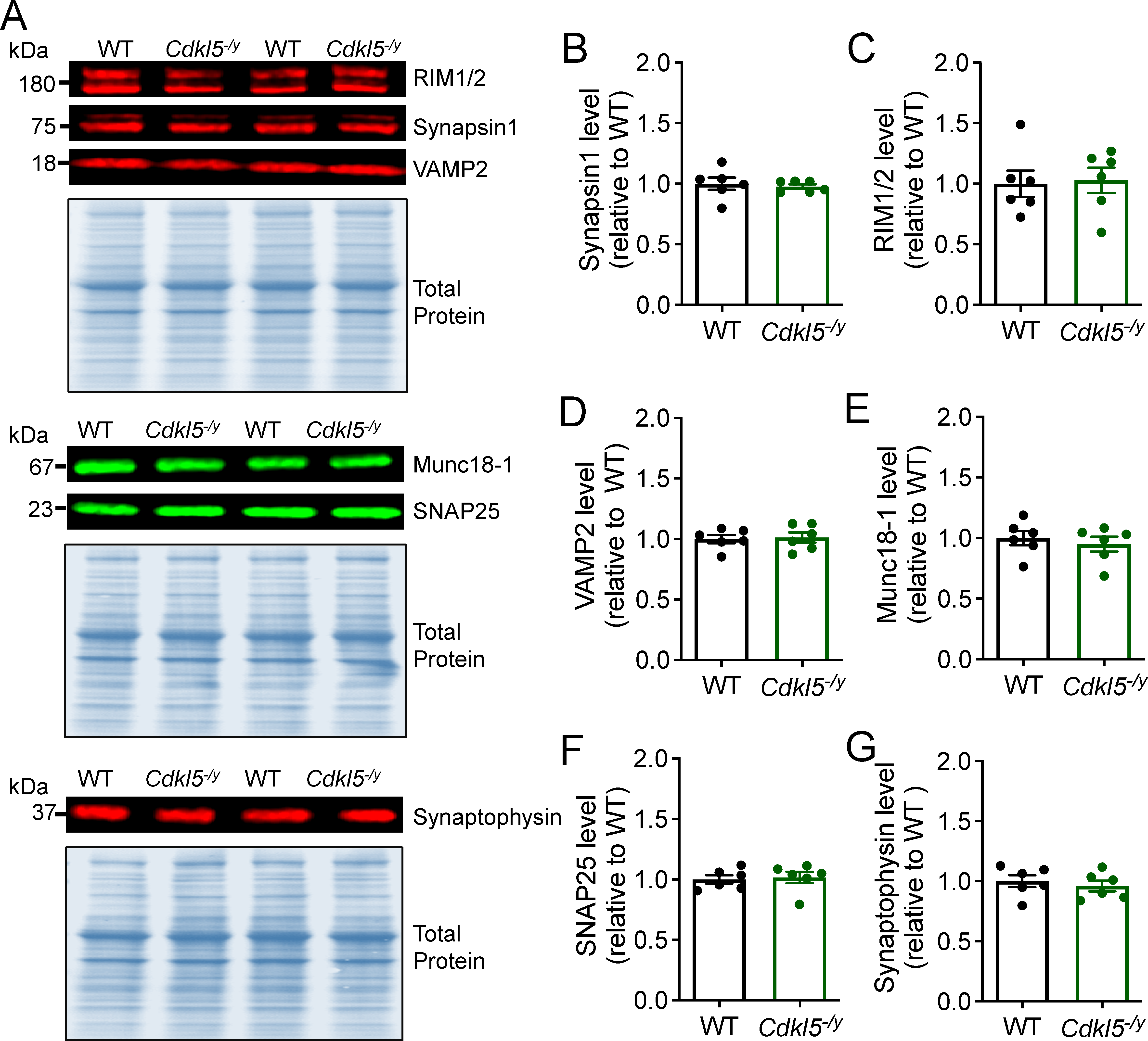

Supplement: Supplementary file 3 — Supplementary Material 3 [file 13229_2024_601_MOESM3_ESM.tif]

Hippocampus P28

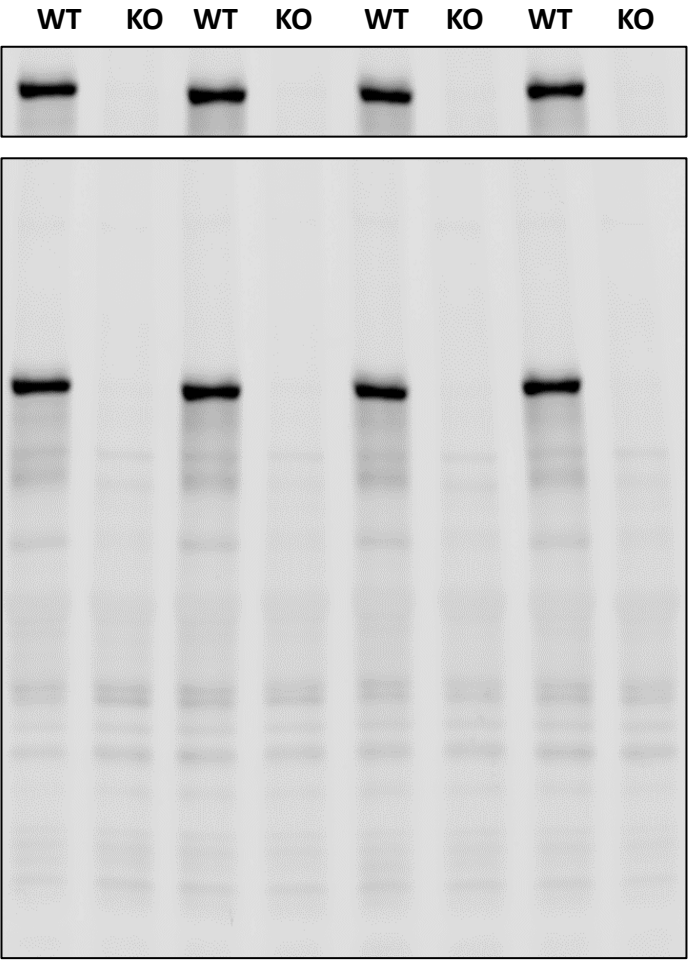

PFC P28

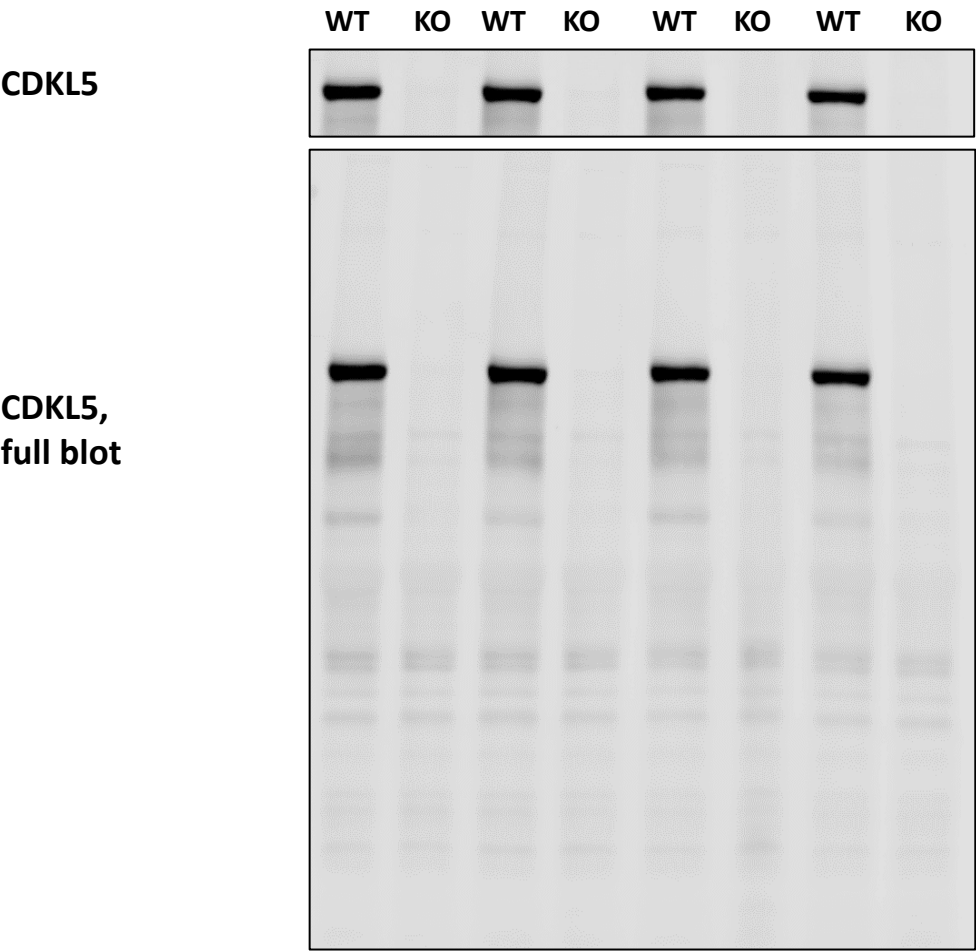

### Hippocampus P28

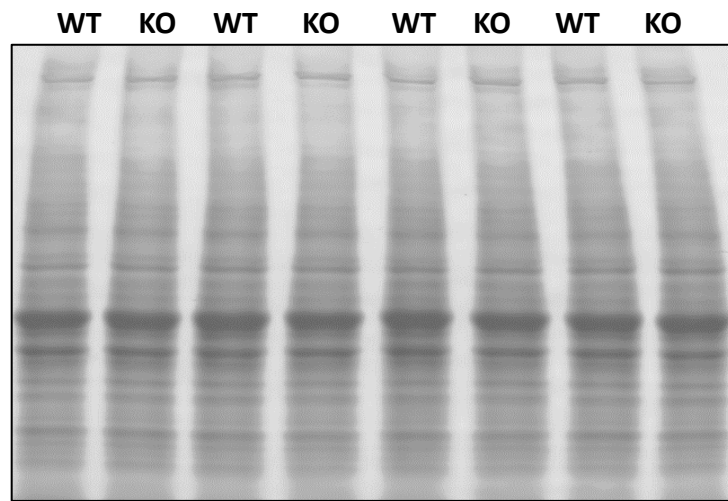

### PFC P28

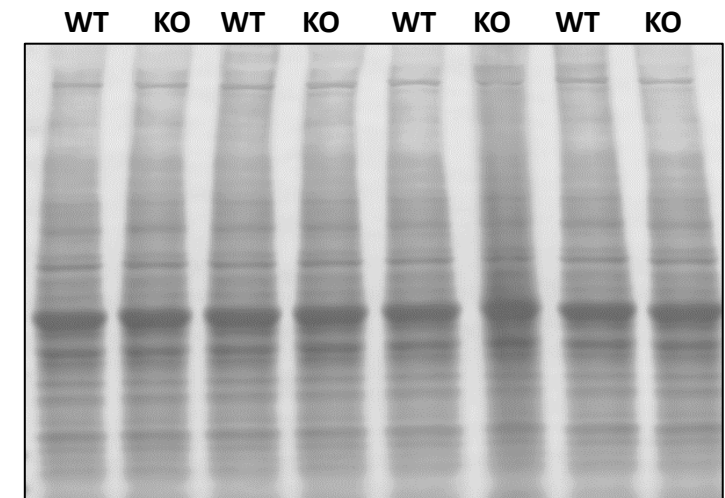

Total Protein

Supplement: Supplementary file 6 — Supplementary Material 6 [file 13229_2024_601_MOESM6_ESM.pdf]
