# Supplementary material for: Enhanced hippocampal LTP but normal NMDA receptor and AMPA receptor function in a rat model of CDKL5 deficiency disorder
Source: Mol Autism. 2024 Jun 14;15:28. doi: 10.1186/s13229-024-00601-9 (PMC11177379; doi:10.1186/s13229-024-00601-9)
Supplement: Supplementary file 4 — Supplementary Material 4 [file 13229_2024_601_MOESM4_ESM.docx]

**Supplemental Figures**

**Enhanced hippocampal LTP but normal NMDA receptor and AMPA receptor function in a rat model of CDKL5 deficiency disorder**

Laura Simões de Oliveira^1, 2^, Heather E O’Leary^3^, Sarfaraz Nawaz^1, 2, 4, 5^, Rita Loureiro^1, 2^, Elizabeth C. Davenport^1^, Paul Baxter^1,6^, Susana R. Louros^1, 2^, Owen Dando^1,2,5^, Emma Perkins^1,2^, Julien Peltier^7^, Matthias Trost^7^, Emily K. Osterweil^1, 2^, Giles E. Hardingham^1, 2, 6^, Michael A. Cousin^1,2,5^, Sumantra Chattarji^1, 2, 4, 5^, Sam A. Booker^1, 2^, Tim A. Benke^3^*, David J. A, Wyllie^1, 2, 5^*, Peter C. Kind^1, 2, 5*^

^1^ Centre for Discovery Brain Sciences, University of Edinburgh, Edinburgh, UK;

^2^ Simons Initiative for the Developing Brain, Patrick Wild Centre, University of Edinburgh, UK;

^3^ University of Colorado, School of Medicine, USA

^4^ National Centre for Biological Sciences, Tata Institute for Fundamental Research, Bangalore, 560065, India

^5^ Centre for Brain Development and Repair, Instem, Bangalore, India

^6^ UK Dementia Research Institute, University of Edinburgh, UK

^7^ Newcastle University Biosciences Institute, Faculty of Medical Sciences, Newcastle upon Tyne, UK, NE2 4HH

* Corresponding authors:

Peter C. Kind

Centre for Discovery Brain Sciences,

Simons Initiative for the Developing Brain,

Hugh Robson Building, University of Edinburgh,

Edinburgh, EH8 9XD, UK

[p.kind@ed.ac.uk](mailto:p.kind@ed.ac.uk)

David J. A. Wyllie

Centre for Discovery Brain Sciences,

Simons Initiative for the Developing Brain,

Hugh Robson Building, University of Edinburgh,

Edinburgh, EH8 9XD, UK

[david.j.a.wyllie@ed.ac.uk](mailto:david.j.a.wyllie@ed.ac.uk)

Tim A. Benke

University of Colorado Denver

Department of Pharmacology

12800 East 19th Ave

Aurora

CO 80045

USA

[tim.benke@cuanschutz.edu](mailto:tim.benke@cuanschutz.edu)


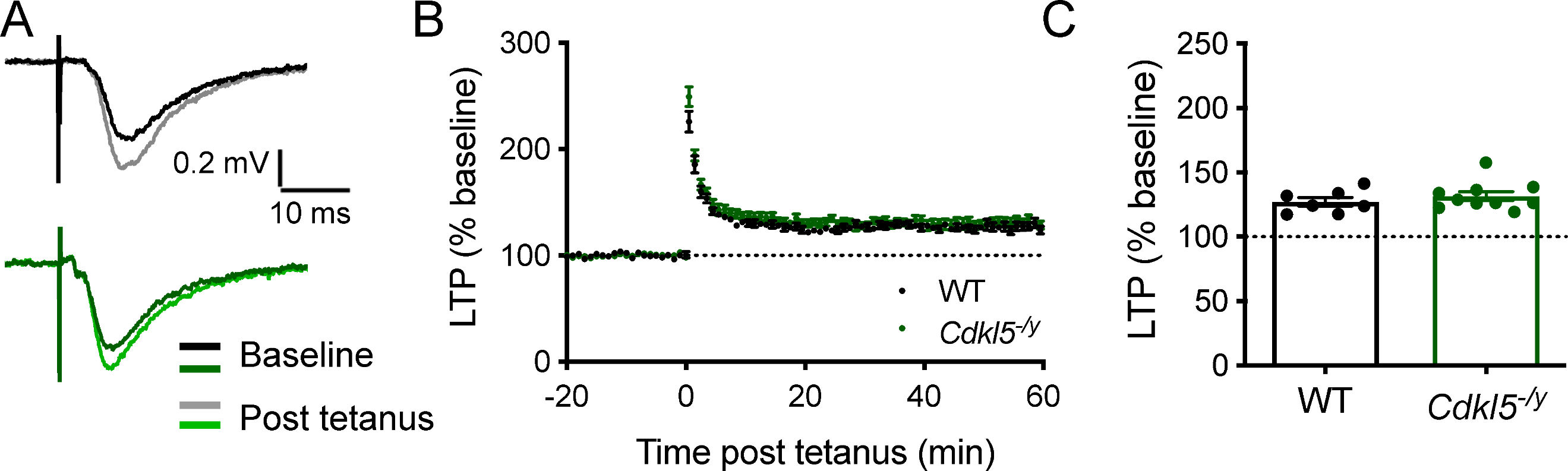


**Supplemental Figure 1. Hippocampal LTP in adult (10-12 weeks) *Cdkl5^-/y^* rats. A -** Representative WT (upper) and *Cdkl5^-/y^* (lower) fEPSP traces before (baseline) and after (post tetanus) LTP induction. **B -** Time-course showing long term potentiation (LTP) in the hippocampal CA1 induced by two trains with 100 pulses at 100 Hz, delivered 20 s apart). **C** – LTP in the final 10 minutes of the recording relative to baseline (WT n = 7 rats; *Cdkl5^-/y^:* n = 9 rats; ns p>0.05 Two tailed T test, data points represent animal averages).


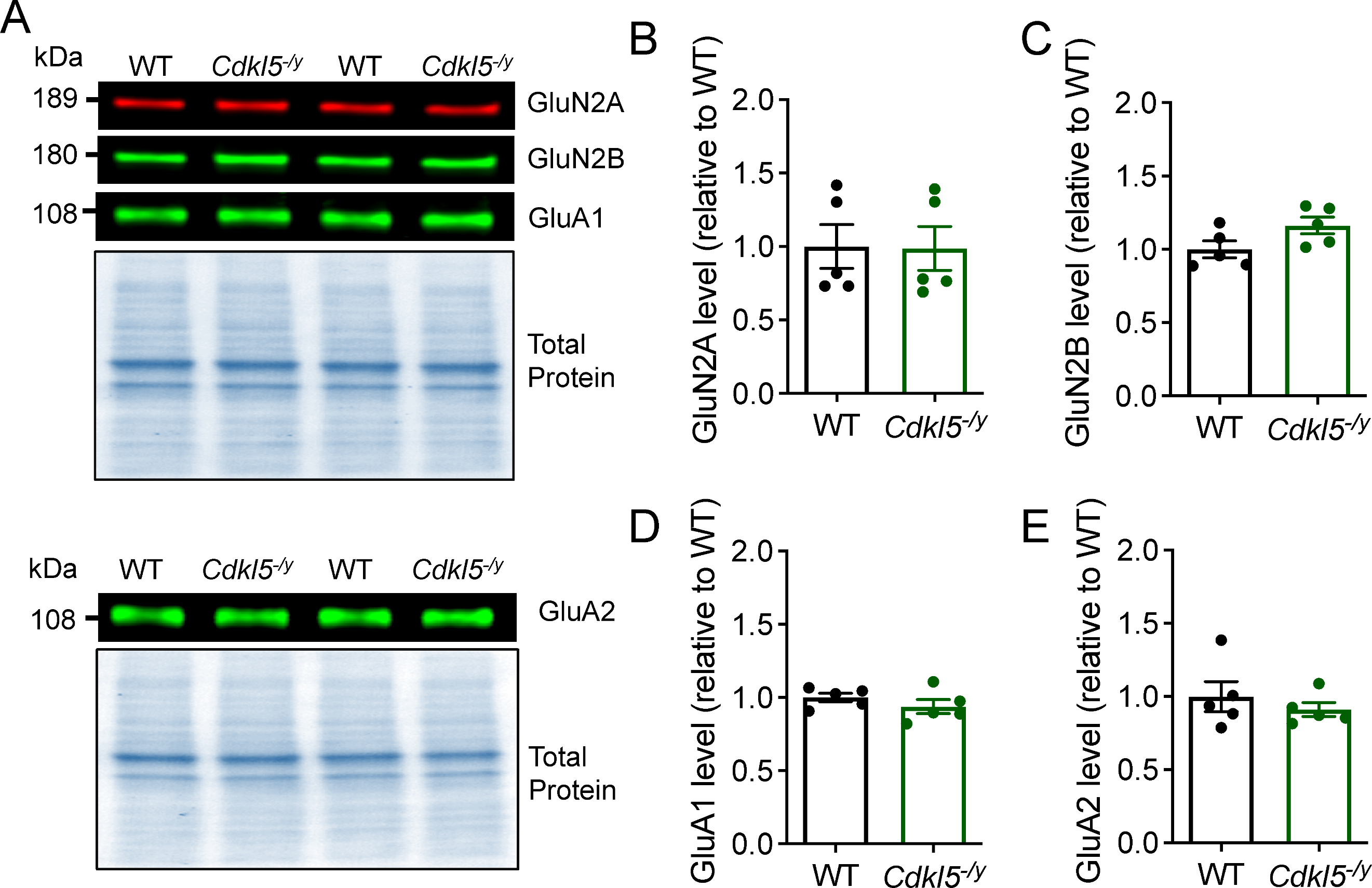


**Supplemental Figure 2. NMDAR and AMPAR subunit expression in hippocampal synaptosome preparations of P14 *Cdkl5^-/y^* rats.** **A** – Representative Western blots for GluN2A, GluN2B, GluA1 and GluA2 with respective Total Protein staining. **B-E** - Quantification of protein expression relative to WT and normalised to total protein (relative to WBs shown in A). All p values > 0.05, WT n = 5 rats; *Cdkl5^-/y^:* n = 5 rats, data shown as mean ± SEM


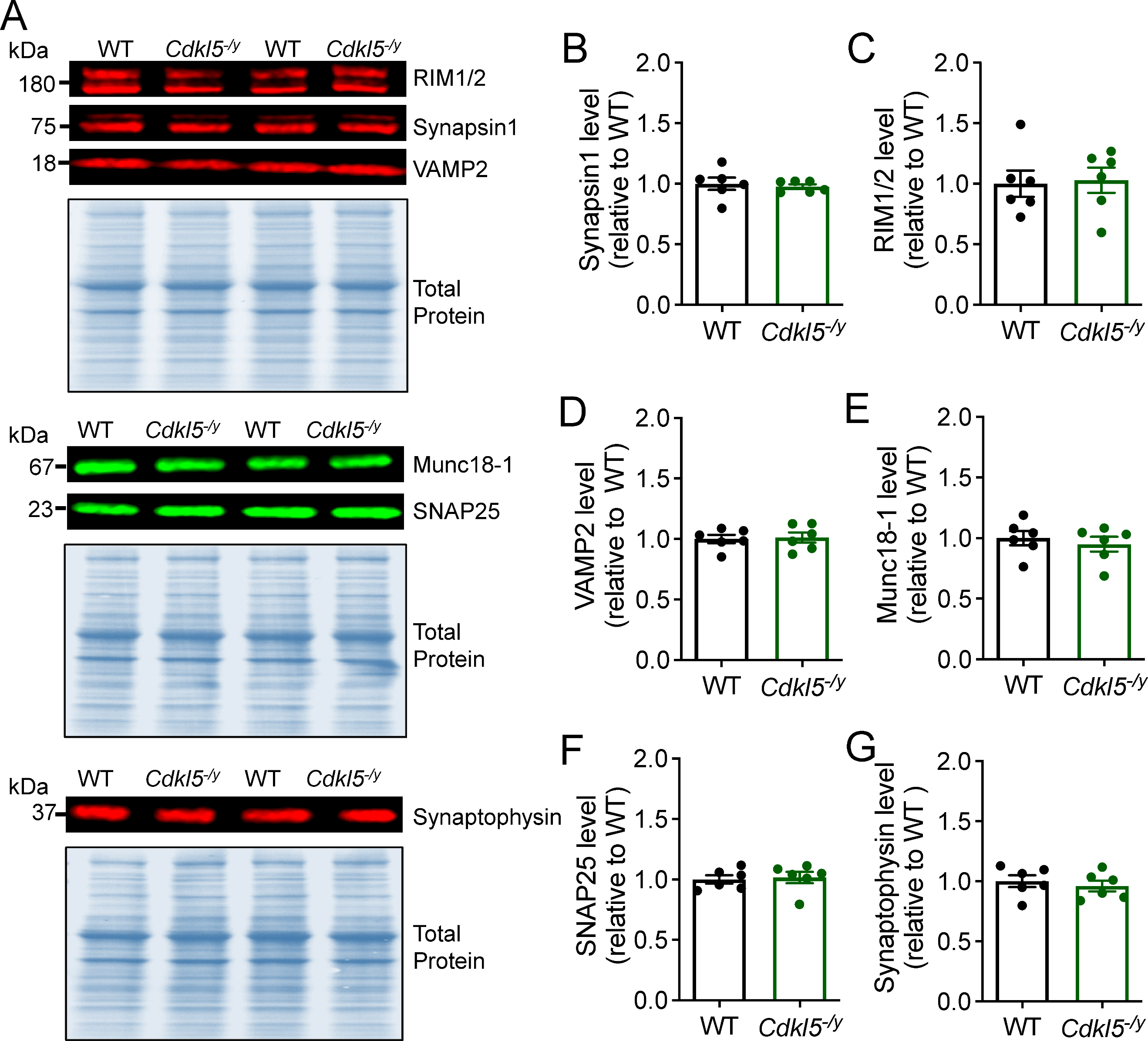


**Supplemental Figure 3. Expression of presynaptic proteins in hippocampal synaptosome preparations of P28-35 *Cdkl5^-/y^* rats.** A – Representative Western blots for presynaptic proteins with respective Total Protein staining. B-G Quantification of protein expression relative to WT and normalised to total protein (relative to WBs shown in A) All p values > 0.05, WT n = 6 rats; *Cdkl5^-/y^:* n = 6 rats, data shown as mean ± SEM.

**Additional File. Table S1.** **Proteomic analysis of *Cdkl5^-/y^* synaptosomes.** Table displays all proteins identified during analysis of both WT and *Cdkl5^-/y^* synaptosomes (Fasta Headers; Column A, Uniprot accession; Column B; Uniprot ID; column C). Column I displays Log2 fold change (*Cdkl5^-/y^* / WT) with proteins that decrease significantly by greater than two fold (equivalent to 1 on log2 scale) in *Cdkl5^-/y^* synaptosomes in green, and proteins significantly increased greater than two fold in orange. Column K reports the P value adjusted for multiple comparisons (significant proteins highlighted in grey).
